# Viral aetiologies of acute encephalitis in a hospital-based South Asian population

**DOI:** 10.1186/s12879-017-2403-z

**Published:** 2017-04-24

**Authors:** Janarthani Lohitharajah, Neelika Malavige, Carukshi Arambepola, Jithangi Wanigasinghe, Ranjanie Gamage, Padma Gunaratne, Pyara Ratnayake, Thashi Chang

**Affiliations:** 1 Department of Pathophysiology, Eastern University, Colombo, Sri Lanka; 2 Department of Microbiology, University of Sri Jayawardenapura, Nugegoda, Sri Lanka; 3 Department of Community Medicine, University of Colombo, Colombo, Sri Lanka; 4 Department of Paediatrics, University of Colombo, Colombo, Sri Lanka; 5 National Hospital of Sri Lanka, Colombo, Sri Lanka; 6 Lady Ridgeway Hospital for children, Colombo, Sri Lanka; 7 Department of Clinical Medicine, Faculty of Medicine – University of Colombo, 25, Kynsey Road, Colombo, 00800 Sri Lanka

**Keywords:** encephalitis, virus, Sri Lanka, South Asia, dengue, WNV

## Abstract

**Background:**

The aetiological spectrum of acute encephalitis shows inter- and intra-geographical variations. We aimed to identify the viruses that cause infectious encephalitis in Sri Lanka, which represents a South Asian population.

**Methods:**

A cross-sectional study was conducted among 99 patients with encephalitis/meningoencephalitis admitted to two tertiary-care hospitals in Colombo. Cerebrospinal fluid and serum were tested for conventional and emerging encephalitogenic viruses. Specific nucleic acid amplification and antibody assays were used to identify viruses. Plaque reduction neutralization test was done to confirm the diagnosis of West Nile virus (WNV).

**Results:**

Patients’ age ranged from 1 month to 73 years (mean = 24.91; SD = 21.33) with a male:female ratio of 1.75:1. A viral aetiology was identified in only 27.3%. These included dengue virus (40.7%), Japanese encephalitis virus (25.9%), varicella zoster virus, WNV and probable Epstein Barr virus (11.1% each). None were positive for herpes simplex viruses or cytomegalovirus. Screening for bacterial aetiologies was negative for all patients. There were no distinguishable clinical or laboratory findings between the different viral aetiologies. The case fatality rate was 7%, which was higher among patients with an identified viral aetiology.

**Conclusions:**

A viral aetiology was identified in only about a quarter of patients with encephalitis. Dengue virus accounted for the majority.

**Electronic supplementary material:**

The online version of this article (doi:10.1186/s12879-017-2403-z) contains supplementary material, which is available to authorized users.

## Background

Encephalitis denotes inflammation of the brain parenchyma that clinically manifests with the syndrome of fever, headache, altered cognition, seizures and focal neurological dysfunction leading to significant mortality and permanent neurological sequelae worldwide [1]. Often the inflammation extends into the meninges causing additional features of meningism and the combined syndrome of meningoencephalitis. The causes of encephalitis are diverse, but broadly categorised as infectious and autoimmune. Viruses are the most commonly identified aetiology of the infectious agents [2], but in up to 70% of cases the cause remains unidentified [3]. However, the recent recognition of several autoimmune encephalitides and use of rigorous diagnostic testing algorithms have substantially decreased the proportion of unknown causes of encephalitis [4].

Of the viruses, herpes simplex virus type 1 (HSV1) is reportedly the commonest cause of adult sporadic encephalitis while varicella zoster virus (VZV) account for most of paediatric encephalitis in developed countries [5, 6]. However, numerically Japanese encephalitis (JE) is the most important global cause of encephalitis causing an estimated 30,000–50,000 cases and about 15,000 deaths annually [7], while West Nile virus (WNV) is the most widespread virus encountered in parts of North America, Europe, Russia, Africa, the Middle East, Indonesia and India [8]. Apart from the established encephalitic viruses, recent studies have identified many emerging encephalitic viruses such as Chandipura and Nipah viruses, particularly in South Asia [9, 10]. However, in Sri Lanka, which is a tropical island positioned between northern latitudes 5° and 10°, the aetiological spectrum of viral encephalitis remains unknown because acute encephalitis surveillance has been confined to JE. This study aimed to identify the viruses causing encephalitis in Sri Lanka to add to the spectrum of encephalitic viruses being identified in South Asia.

## Methods

### Patients and samples

Using a cross-sectional study design, consecutive patients with a clinical syndrome of encephalitis/meningoencephalitis admitted to the two largest tertiary-care hospitals in Sri Lanka for adults (National Hospital of Sri Lanka, bed-strength 3900) and children (Lady Ridgeway Hospital for Children, bed-strength 900) between November 2012 and August 2014 were recruited. The syndrome of encephalitis/meningoencephalitis was defined based on the Consensus Statement of the International Encephalitis Consortium [11] as depressed or altered level of consciousness, change in behaviour or personality lasting for ≥24 h and requiring hospitalization for one or more of the following symptoms: fever, seizures or focal neurological dysfunction. Patients with an alternative diagnosis that could mimic encephalitis such as psychiatric illness, metabolic disorders, epilepsy, post-anoxia, vasculitis, stroke and septicaemia, and patients in whom lumbar puncture was contraindicated were excluded. Diagnosis of encephalitis was made by a Board-certified specialist in Neurology, Paediatric Neurology, Internal Medicine or Paediatrics. The computed minimum sample size to achieve a precision of 0.05 and Z_α_ value of 1.96 was 87. Written informed consent was obtained from either the patient or guardian. Serum and/or cerebrospinal fluid (CSF) were obtained from all patients when these specimens were collected as part of their diagnostic work up. Demographic, clinical and laboratory data including CSF analysis, blood counts, brain imaging and electroencephalogram (EEG) results were recorded. The paediatric age group was defined as less than 12 years.

### Laboratory analysis

PCR assays were performed for EBV, CMV, VZV, HSV, WNV, JEV and dengue while IgM and IgG antibody tests were performed for HSV, JEV, WNV and dengue in serum and CSF (when available) in all patients (*n* = 99).

#### Nucleic acid extraction

Nucleic acid was extracted from 200 μl (for DNA) and 140 μl (for RNA) of clinical specimens (blood/serum, CSF) using the spin protocol of QIAamp DNA mini blood kit (QIAGEN**®**) and QIAamp viral RNA mini kit (QIAGEN**®**) and stored at -80 °C until further analysis. For DNA extraction from CSF carrier DNA, poly dA (Promega, USA) was used.

#### PCR and RT-PCR analysis

Polymerase chain reaction (PCR) was used to detect DNA viruses such as herpes simplex virus type 1 (HSV-1) and type 2 (HSV-2), varicella zoster virus (VZV), Epstein Barr virus (EBV) and cytomegalovirus (CMV). Reverse transcriptase polymerase chain reaction (RT-PCR) was used to detect RNA viruses such as dengue and Japanese encephalitis virus. For primers used and their target genes see Additional file 1.

#### PCR

DNA samples were amplified in a 50 μl PCR reaction as previously described [12–15]. DNA extracted from clinical specimens of patients who were known to be having acute infections of herpes simplex, varicella, CMV and EBV were used as positive controls and deionised water was used as negative control.

#### RT-PCR

RNA samples were amplified in a 50 μl RT-PCR reaction mixture as previously described [16]. RNA extracted from serum of dengue infected patients and RNA from JE vaccine were used as positive controls for dengue and JE RT-PCR, respectively. Deionized water was used as negative control. PCR products were separated on 2% *w*/*v* agarose (Promega) in 1*TAE buffer.

#### ELISA

Enzyme-linked immunosorbant assays (ELISA) were used to detect specific immune reactions utilizing IgM capture ELISA for HSV-1, HSV-2 (Abcam, UK), dengue (Panbio), JEV (*InBiOS*, USA), WNV (*InBiOS*, USA) and IgG capture ELISA for HSV-1, HSV-2 (Abcam,UK) and Dengue virus (Panbio). Dengue NS1 antigen was also tested (dengue early ELISA, Panbio). The results were interpreted according to the manufacturer’s instructions.

#### Bacterial screening

CSF Gram stain, culture and blood cultures were performed to exclude bacteriological infection. Tuberculosis is notoriously difficult to isolate in central nervous system disease. However, this was excluded based on the clinical syndrome, inflammatory markers, neuroimaging and CSF profiles and staining for acid fast bacilli, and when indicated, culture and PCR for *Mycobacterium tuberculosis*.

#### Confirmatory test

Plaque reduction neutralization test (PRNT) was done at the National WNV reference laboratory of the National University of Singapore only for samples that were positive for WNV-specific IgM.

### Definitions for diagnosis

JEV encephalitis was diagnosed as definite if RT-PCR for JEV was positive or if JEV-specific IgM was detected in CSF and the ISR value for JEV-specific IgM was over two-fold more than the IgM index value for dengue and ISR value for WNV.

Dengue encephalitis was diagnosed as definite if RT-PCR for DENV was positive or if DENV-specific IgM was detected in CSF. Dengue encephalitis was considered probable if DENV-specific IgM was not detected in CSF, but detected only in serum.

WNV encephalitis was diagnosed as definite if WNV-specific IgM was detected in CSF and WNV neutralizing antibodies were detected on PRNT.

VZV encephalitis was diagnosed as definite if VZV-PCR was positive in CSF or in serum. EBV encephalitis was diagnosed as probable if EBV-specific DNA was detected in CSF since EBV-specific antibody tests were not performed to differentiate between acute and reactivated infection.

### Ethical considerations

The study was approved by the Ethics Review Committees of the Faculty of Medicine, University of Colombo, Sri Lanka and that of the two hospitals. Informed written consent was obtained from the patient, or from the guardian in the case of mentally incompetent patients or children. None refused consent to participate.

## Results

**Fig. 1:**
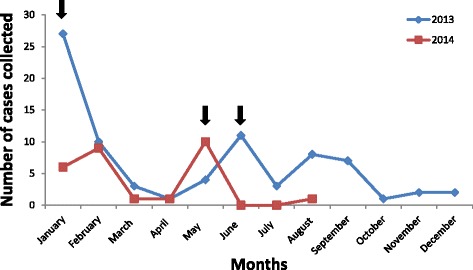
Temporal distribution of cases of encephalitis recruited during the study period. Arrows indicate peak periods of highest frequencies

A total of 108 patients were recruited over a period of 21 months, but 9 were subsequently excluded due to inadequate clinical material for analysis. Patients’ age ranged from 1 month to 73 years (mean = 24.91; SD = 21.33) with 41.4% being less than 12 years. The male to female ratio was 1.75:1 in both adults and children. Patients were residents from 16 of the 25 administrative districts in Sri Lanka. Most cases peaked during the months of January/February and May/June consistent with the temporal pattern of encephalitis noted in Sri Lanka (Fig. 1).

The clinical and laboratory characteristics of patients are shown in Table 1. Brain imaging had been performed with computerised tomography (CT) in 59 patients and magnetic resonance imaging (MRI) in 31 patients. Abnormalities were noted in 65.5% of CT scans and 67.8% of MRI scans. The most common of the neuroimaging abnormalities was focal or generalised cerebral oedema (76.9%) on CT followed by focal cerebritis (36.8%), cerebral infarction (21.1%) and meningeal enhancement (15.8%) on MRI. None of the imaging abnormalities were specific to any of the identified virus types. EEGs had been done in 33 patients and were abnormal in 72.4%.

**Table 1:** Clinical and laboratory characteristics of patients with encephalitis

Clinical / laboratory characteristic	Number of patients (of *n* = 99)
Altered level of consciousness	99
Fever	93
Headache	54
Seizures	58
Prodromal symptoms	77
Focal neurological signs	44
Cerebellar signs	12
Extrapyramidal signs	6
Neuropsychiatric symptoms	21
Cerebrospinal fluid	
Protein >40 mg/dl	22
CSF:plasma glucose ratio < 30%	39
Pleocytosis	26
Gram stain positivity	0
Bacterial culture positivity	0

A viral aetiology was identified in only 27.3%, more among adult (38%) than paediatric patients (12.2%). Results of viral detection using IgM capture ELISA and PCR are shown in Table 2. The viral aetiologies identified included dengue virus (40.7%), JE virus (25.9%), VZV (11.1%) and WNV (11.1%). Two of the three patients positive for WNV specific IgM were tested for PRNT50 neutralizing antibodies and were found to have titres of 800 against WNV confirming definite WNV infection. Since EBV was detected only by PCR of CSF without serological confirmation, its role as an aetiological agent was considered probable and not definite (see ‘Definitions’ above). None of the patients were positive for HIV.

**Table 2:** Viruses identified in serum and/or CSF using polymerase chain reaction and IgM capture ELISA

Viruses	Number of patients positive of 99 tested		Total
	PCR / RT-PCR	IgM - ELISA	
Varicella zoster virus	03	ND	03
Epstein Barr virus	03	ND	03
Cytomegalovirus	00	ND	00
Herpes simplex types 1 and 2	00	00	00
Japanese encephalitis virus	00	07	07
Dengue virus	00	11	11
West Nile virus	00	03	03
Total	06	21	27

*ND* Not Done

There were no distinguishable clinical or laboratory characteristics between the different viral aetiologies (Table 3). Although dengue fever is characterised by fever and thrombocytopenia, which can be complicated by extravascular fluid leakage, haemorrhage and hepatitis, among patients with dengue virus encephalitis, only 4 of 11 had thrombocytopenia while none had evidence of fluid leakage, haemorrhage or hepatic necrosis. Seven patients with dengue virus encephalitis developed seizures. Four of 7 patients with JE developed extrapyramidal features and neuropsychiatric manifestations. None of the other aetiologies manifested extrapyramidal features. Only 1 of the 3 patients with WNVE had CSF neutrophil pleocytosis. Two of the three patients with VZV encephalitis developed seizures while one had features of cerebellitis, but none developed a skin rash. All three patients with probable EBV encephalitis developed seizures while one had periodic lateralising epileptiform discharges on EEG.

**Table 3:** Clinical and laboratory characteristics of patients with encephalitis according to aetiological diagnosis

Encephalitis aetiology	Seizures	Focal neurological signs	Neck stiffness	Headache	Neuro-psychiatric features	Extra-pyramidal features	Cerebellar signs	Thrombo cytopaenia	CSF pleocytosis	Outcome
DV1	−	−	+	+	−	−	−	−	Lymphocytic	Discharged
DV2	−	−	−	+	−	−	−	+	None	Discharged
DV3	−	−	+	+	−	−	−	−	None	Discharged
DV4	+	+	+	−	−	−	−	−	None	Death
DV5	+	−	−	+	−	−	−	+	None	Death
DV6	+	+	−	−	−	−	−	+	None	Discharged
DV7	+	−	+	+	−	−	−	−	None	Discharged
DV8	+	−	+	+	−	−	−	−	Lymphocytic	Discharged
DV9	+	+	−	−	−	−	−	−	None	Discharged
DV10	+	−	−	−	Abnormal behaviour	−	−	−	None	Discharged
DV11	−	+	−	−	−	−	−	+	None	Discharged
JE1	+	+	−	−	Hallucination	+	−	−	None	Discharged
JE2	−	+	+	+	−	−	−	−	ND	Death
JE3	−	+	+	−	−	−	−	−	Lymphocytic	Discharged
JE4	−	+	+	+	Aggressive	+	−	−	None	Discharged
JE5	−	−	+	−	Hallucination	−	−	−	None	Discharged
JE6	−	+	−	+	Hallucination	+	−	−	None	Discharged
JE7	+	+	+	−	−	+	−	−	None	Discharged
WNV1	+	−	−	−	−	−	−	−	None	Discharged
WNV2	−	−	+	+	−	−	−	−	Neutrophilic	Discharged
WNV3	+	−	+	+	−	−	−	−	Lymphocytic	Discharged
VZV1	+	+	+	−	−	−	+	−	None	Discharged
VZV2	+	−	−	−	−	−	−	−	None	Discharged
VZV3	−	−	−	+	−	−	−	−	Lymphocytic	Discharged
EBV1	+	+	+	+	−	−	+	−	Lymphocytic	Discharged
EBV2	+	+	−	−	−	−	−	−	None	Death
EBV3	+	+	−	−	−	−	−	−	Lymphocytic	Death

All patients had altered level of consciousness and fever

*DV* Dengue virus, *JE* Japanese encephalitis, *WNV* West Nile virus, *VZV* Varicella zoster virus, *EBV* Epstein Barr virus, *M* male, *F* female, *CSF* cerebrospinal fluid, *EEG* electroencephalogram, *MRI* magnetic resonance imaging, *CT* computerised tomography, *ND* not done

The mean duration of hospital stay was 15 days (SD 12.5; range 2–90). All patients were treated with intravenous aciclovir and varying courses of antibiotics and steroids. Death occurred in six adults and one child resulting in a case fatality rate of 7% in this study. The mortality rate was higher among patients in whom a viral aetiology was identified (5/27) than in patients in whom an aetiology was not identified (2/72) (Table 3). This difference was statistically significant (*p* < 0.05).

## Discussion

Despite a wide array of stringent laboratory testing, a viral aetiology was identified in only just over a quarter of patients presenting to hospital with a syndrome of encephalitis/meningoencephalitis. Dengue virus accounted for 41% while Japanese encephalitis accounted for 26%. We identified three patients with WNV neuroinvasive disease. This was the first identification of human WNV in Sri Lanka, which was reported by us earlier because of its public health implications [17]. It is likely that a proportion of ‘virus-negative’ patients had autoimmune encephalitis or encephalitis due to emerging viruses or viruses not tested in our study.

In Sri Lanka, surveillance of encephalitis is confined to JE, which has been endemic for several decades and caused epidemics in the mid and late 1980s [18]. However, JE accounts for only 39% of all notified cases of acute encephalitis in Sri Lanka (Epidemiological Bulletin 2012, Epidemiology Unit, Sri Lanka), and the aetiological spectrum of viral encephalitis has remained unknown. Given that HSV and VZV have been reported as the commonest viral aetiologies of encephalitis in developed countries, it has been usual practice to administer intravenous aciclovir to all patients presenting with suspected infectious encephalitis in Sri Lanka. However, none in our study was positive for HSV. This may have been partly related to the low HSV seroprevalence in Sri Lanka. For example, the HSV-1 seroprevalence was 74.4% among individuals aged 35 to 40 years who attended sexually transmitted diseases clinics in Sri Lanka compared to a seroprevalence of over 90% in the general population in most other countries [19], and partly related to the timing of specimen collection, since it has been observed that the yield of PCR for HSV is less during the first 3 days and after 10–14 days of disease onset [20]. For this reason, and because we detected VZV sans skin manifestations in our study, we would recommend continuing the practice of empirical intravenous aciclovir in acute encephalitis unless an alternative aetiology is evident.

Dengue virus is endemic in Sri Lanka and has emerged as the most important vector-borne disease causing recurrent epidemics [21]. Dengue is recognized as a frequent or leading cause of encephalitis in endemic regions and dengue encephalitis may be the primary manifestation of infection [22, 23]. Given that there were 32,063 cases of dengue reported in Sri Lanka during the period that this study was conducted, it is not surprising that dengue has emerged as the commonest viral aetiology in our study. Although thrombocytopaenia associated with extravascular fluid leakage are highly suggestive of dengue infection in endemic regions, in our study, dengue encephalitis occurred as a primary manifestation of dengue infection with only a third of dengue encephalitis patients developing thrombocytopaenia while none had fluid leakage.

Six of the seven patients with JE were over the age of 20 years. It is likely that none of these patients had been immunised given that immunisation began in a phased basis in 1988 and reached country-wide coverage in only 2011. Flaccid paralysis in the context of encephalitis suggests the aetiologies of JE and WNV. However, none of our patients demonstrated this feature, even among those diagnosed with JE and WNV. Notably, extrapyramidal features were noted in only JE while neuropsychiatric manifestations occurred predominantly also in JE. However, extrapyramidal features and neuropsychiatric manifestations are known to occur in other forms of encephalitis, particularly autoimmune encephalitis [24, 25].

This study resulted in the identification of human WNV in Sri Lanka, which has the requisite environment for the maintenance of the zoonotic WNV life-cycle between the *Culex* genus of mosquitoes and passerine birds. However, there were no distinguishing clinical characteristics in the WNV infected patients [17]. The presence of neutrophil predominance in CSF has been suggested as a possible diagnostic clue of WNV infection [26], but only one of our three patients had CSF neutrophilic pleocytosis.

Although VZV had been identified as a common aetiology among paediatric encephalitis in developed countries [5], all 3 VZV patients in our study were adults. It is likely that our patients represented reactivated disease while those reported in developed countries were related to primary infection. Furthermore, compared to developed countries where 95% of the population are seropositive for VZV at 5 years, in Sri Lanka it is predominantly an adult disease with seroprevalence reaching 50% at 60 years in rural populations [27]. VZV vaccine in not routinely given to children in Sri Lanka. Moreover, chickenpox has been noted to be more common in adult than paediatric populations in Sri Lanka [28]. Enteroviruses have now superseded VZV as commonest cause of childhood encephalitis in developed countries, likely because of better use of molecular tests, and because of implementation of VZV vaccines [29]. Conspicuously, none of our VZV encephalitis patients manifested a skin rash highlighting primary CNS presentation of the virus.

Chandipura and Nipah viruses have been recognised as emerging viral aetiologies of several outbreaks of encephalitis in South Asia [9, 10] and Chandipura virus has been isolated from toque macaque monkeys in Sri Lanka [30]. Although we did not detect either of these viruses in our study population, these viral aetiologies were not completely excluded since we lacked appropriate positive controls for these viruses in our assays.

The mortality rate was lower among patients with an unidentified aetiology. This may suggest less virulence of agents not screened in our panel of attributable viruses or self-limiting autoimmune aetiologies.

The strengths of our study stems from the inclusion of both paediatric and adult patients admitted to the two largest tertiary referral centres in a relatively small country which facilitates referral of patients to these centres from many parts of the country as reflected by the distribution of our patients. Furthermore, encephalitis is a serious neurological disorder that invariably presents to hospital, which justifies studying a hospital-based population. We used both serology and nucleic acid techniques rigorously in attempting to identify an infectious aetiology. Weaknesses include the lack of appropriate positive controls for assays of Nipah and Chandipura virus detection and the lack of serological confirmation of EBV. Furthermore, our aetiological analysis did not include all viruses that can potentially cause encephalitis and hence, a proportion of patients with a viral aetiology may have been missed. A further weakness was that our study did not include long-term follow-up of patients.

## Conclusions

The causes of infectious encephalitis are many and varied, and accurate diagnosis is necessary for appropriate treatment and accurate prognostication. Our study is consistent with previous observations in the elusiveness of identifying aetiological agents of encephalitis [31], but highlights the need to widen the net to capture emerging agents.

## Additional file

Additional file 1: Primers and their target genes. (DOCX 14 kb)

## Abbreviations

CMV: Cytomegalovirus
CSF: Cerebrospinal fluid
CT: Computerised tomography
EBV: Epstein Barr virus
EEG: Electroencephalogram
ELISA: Enzyme-linked immunosorbant assay
HSV: Herpes simplex virus
ISR: Immune status ratio
JEV: Japanese encephalitis virus
MRI: Magnetic resonance imaging
PCR: Polymerase chain reaction
PRNT: Plaque reduction neutralization test
RT-PCR: Reverse transcriptase polymerase chain reaction
VZV: Varicella zoster virus
WNV: West Nile virus
