# Supplementary material for: Viral aetiologies of acute encephalitis in a hospital-based South Asian population
Source: BMC Infect Dis. 2017 Apr 24;17:303. doi: 10.1186/s12879-017-2403-z (PMC5404678; doi:10.1186/s12879-017-2403-z)
Supplement: Additional file 1: — Primers and their target genes. (DOCX 14 kb) [file 12879_2017_2403_MOESM1_ESM.docx]

| **Virus** | **Gene** | **Primer sequence (5’to3’)** |
| --- | --- | --- |
| **HSV1 & 2** | DNA polymerase | PL: ATCAACTTCGACTGGCCCTTC |
|  |  | PR: CCGTA CATGTCGATGTTCACC |
|  | gB | Sense: GCATCGTCGAGGAGGTGGAC |
|  |  | Antisense:TTGAAGCGGTCGGCGGCGTA |
| **VZV** | ORF 38 | Forward: AAGTTTCAGCCAACGTGCCAATAAA |
|  |  | Reverse: AGACGCGCT TAACGGAAGTAACG |
| **CMV** | gB | Sense: ACGACCCGTGGTCATCTT TA |
|  |  | Antisense: GCGGTGGTTGCCCAACAGGA |
| **EBV** | BamHI-W | Primer 1: TCGCGTTGCTAGGCCACCTT |
|  |  | Primer 2: CTTGGATGGCGGAGTCAGCG |
| **Dengue virus** | Genome position 134 - 161  616 – 644 | D1: TCAATATGCTGAAACGCGCGAGAAACCG |
|  |  | D2: TTGCACCAACAGTCAATGTCTTCAGGTTC |
| **JEV** | Premembrane gene | Forward: CGTTCTTCAAGTTTACAGCATTAGC |
|  |  | Reverse: CCYRTGTTYCTGCCAAGCATCCAMC |

**Additional file 1**

**Primers and their target genes**
